# Photobiomodulation in Brazil: Current scenario and evidence-based practice in the voice clinic

**DOI:** 10.1016/j.bjorl.2024.101543

**Published:** 2024-12-14

**Authors:** Gustavo Polacow Korn, Renata Rangel Azevedo, Viviane Souza Bicalho Bacelete, Claudia Alessandra Eckey

**Affiliations:** a Universidade Federal de São Paulo, São Paulo, SP, Brazil; Universidade Federal de Minas Gerais, Belo Horizonte, MG, Brazil; Fleury Medicina e Saúde Laboratórios de Diagnóstico, São Paulo, SP, Brazil

The voice clinic increasingly uses photobiomodulation as an adjuvant resource to enhance therapeutic gains. Even though cell photobiomodulation mechanisms have not been fully understood yet, the most accepted theory is that low-level light therapy works on tissues, reducing the inflammatory process, increasing cell bioenergetics through its direct action on mitochondria, and increasing ATP production.^1^ The few studies on the topic published so far suggest an enhanced vocal fold healing process, anti-inflammatory potential, and effects on vocal fatigue.^2^, ^3^, ^4^, ^5^

There is a clinical impression of the therapeutic benefits of photobiomodulation on voice, but few scientific studies provide evidence-based practice guidelines supporting this thesis. Three studies have shown promising results with this therapy, one of which treats laryngopharyngeal reflux induced by intubation in mice,^2^ one *in vitro* study with lamina propria epithelial cells of one person’s vocal folds,^3^ and another *in vivo* study with rabbits’ vocal folds for healing analysis.^4^ Results suggest that photobiomodulation may inhibit the inflammatory reaction, enhance the secretion of hyaluronic acid, decrease collagen deposition, and increase cell proliferation and migration in vocal folds and genes involved in the healing process. Kagan and Heaton (2017), in a prospective randomized study with 16 adults, observed an improvement in vocal fatigue with this therapy, suggesting the need for future studies to determine wavelengths and more efficient doses.^5^

Therefore, despite being widely used in the voice clinic, there is no robust evidence of its effects as a therapeutic technique, considering aspects of clinical protocol such as dosimetry parameters, irradiated area, and time-response factor. Since selecting irradiation parameters often poses a dilemma, it is desirable to standardize anatomical points and identify possible optimum biomodulation doses for better decision-making. The limited scientific evidence and potential side effects have raised concerns among physicians and speech-language-hearing pathologists dedicated to the voice clinic.

The way low-level laser therapy works also needs further clarification. Are the effects observed in clinical practice due to changes in vocal muscles and/or other intrinsic laryngeal muscles? Or extrinsic muscles? And what is the effect of this therapy on the vocal fold lamina propria? Possible adverse events of using this therapy should also be considered. The larynx is remarkably close to the thyroid gland, and the possible effects on this gland are unknown in the short, medium, and long term. The recommended application technique involves proper neck palpation and the correct identification of laryngeal anatomical points, ensuring safe and precise irradiation on the target area. Plec *et al*^6^ have described laryngeal anatomical points for irradiation and found differences in laser light penetration according to skin phototype and body mass index. The neck is highly vascularized, requiring knowledge of the patient’s history and physiological anatomy before this procedure. Professionals should also request additional exams since vocal fold hemorrhage, neoplasms, papilloma, leukoplasia, or any thyroid condition call for cautious use, and may often contraindicate irradiation. Would it be interesting to assess the thyroid – e.g., a thyroid function test and an ultrasound – before beginning this therapy? It is important to consider prior medical assessments to rule out premalignant and malignant laryngeal lesions and thyroid nodules since the effect on these conditions has not yet been clarified. The interaction of light with biological tissues depends on personal characteristics (age, sex) and tissue peculiarities (cutaneous phototype, subcutaneous fat, physiological state of the tissue, types of cells, and blood perfusion). These factors increase the complexity of dosimetry and photobiomodulation, and light-tissue interactions must be better understood to optimize therapeutic techniques. Basic photobiomodulation science requires knowledge of several areas, such as chemistry, physics, and biology. Hence, such complex biological processes cannot be simplified without a solid basis.

Delivering energy in deeper tissues must overcome several barriers, and the larynx is an anatomical complex involving cartilage, muscles, and membranes. Thus, what is the ideal light wavelength to overcome all anatomical barriers and interact with laryngeal structures? What energy is required for absorption and photochemical reactions at several depths? It is greatly important to approach the optical properties of the tissue to preview therapeutic light distribution and energy absorption. Would it not be important to establish a therapeutic irradiation window considering necks of different diameters, fat composition, and different cartilage (men/women, younger/older people, and individuals with more or less melanin)?

The literature eloquently points out that despite significant continuous progress and advances in photobiomodulation, inconsistent clinical outcomes have posed conflicts in light therapy use. For this reason, standardization and rigorous analyses of irradiation parameters are extremely valuable to improve clarity and boost critical progress in the field, moving photobiomodulation toward legitimacy.

It is extremely important to develop accurate protocols that include individual characteristics aligned with the proper wavelength, dose, power density or irradiance, energy density or fluence, depth of tissue treated, irradiation time, size of the beam point reaching the surface of the target tissue, and the periodicity and number of sessions to avoid controversy and empiricism in conclusions.

We hope this editorial stimulates research based on future guidelines grounded on the best scientific evidence so that light therapy may be applied safely and assertively in the voice clinic once its benefits and risks are known.

## Funding

None.

## Conflicts of interest

The authors declare no conflicts of interest.
